# Adenosine receptor expression in rheumatoid synovium: a basis for methotrexate action

**DOI:** 10.1186/ar3871

**Published:** 2012-06-08

**Authors:** Lisa K Stamp, Jody Hazlett, Rebecca L Roberts, Christopher Frampton, John Highton, Paul A Hessian

**Affiliations:** 1 Department of Medicine, University of Otago, Christchurch, 2 Riccarton Ave, Christchurch 8014, New Zealand; 2 Department of Physiology, University of Otago, Great King Street, Dunedin 9016, New Zealand; 3 Department of Biochemistry, University of Otago, Great King Street, Dunedin 9016, New Zealand; 4 Department of Medicine, University of Otago, Great King Street, Dunedin 9016, New Zealand

## Abstract

**Introduction:**

Methotrexate (MTX) exerts at least part of its anti-inflammatory effects through adenosine receptors (ADOR). The aims of this study were to determine the expression of all four adenosine receptor genes (ADORA_1_, ADORA_2A_, ADORA_2B_, ADORA_3 _and ADORA_3variant_) in rheumatoid synovial tissue and any influence of MTX exposure on this expression. Furthermore, we investigated whether polymorphisms within ADORA_3 _were associated with response and/or adverse effects associated with MTX.

**Methods:**

Adenosine receptor gene expression was undertaken using PCR in 20 rheumatoid arthritis (RA) synovial samples. A separate cohort of 225 RA patients receiving MTX was genotyped for SNPs in the ADORA_3 _receptor gene. Double immunofluorescence was used to identify cells expressing ADOR protein.

**Results:**

All ADOR genes were expressed in all synovial samples. ADORA_3 _and A_3variant _were the dominant subtypes expressed irrespective of MTX therapy. Expression of ADORA_2A _and ADORA_2B _was increased in patients receiving MTX compared to those not receiving MTX. There was no association between the ADORA_3 _*rs1544224 *SNP and high and low disease activity or MTX-associated adverse effects. ADORA_2B _protein expression was most obvious in vascular endothelial cells whereas ADORA_3 _protein was more abundant and expressed by synovial fibroblasts.

**Conclusions:**

We have shown that adenosine receptors are expressed in RA synovium. There is differential expression of receptors such that ADORA_3 _is expressed at significantly higher levels. This evidence demonstrates the potential for MTX to exert its anti-inflammatory effects at the primary site of pathology within the joints of patients with RA.

## Introduction

Methotrexate (MTX) remains the first line drug for the treatment of rheumatoid arthritis (RA). The exact mechanism of action of MTX is complex. Following administration and absorption, serum MTX concentrations fall rapidly [1] as MTX is transported into a variety of cells including red blood cells (RBC) and synoviocytes. Intra-cellularly, glutamate moieties are added by folylpolyglutamate synthetase (FPGS) such that MTX is retained within cells as MTX polyglutamates (MTXGlu_n_).

MTX polyglutamates inhibit a number of important intracellular enzymes in the folate pathway including dihydrofolate reductase and thymidylate synthase. Inhibition of 5-aminoimidazole-4-carboxamide ribonucleotide (AICAR) transformylase (ATIC) results in accumulation of AICAR and increased adenosine release into the circulation. Extracellular adenosine increases cAMP which inhibits production of pro-inflammatory cytokines including TNF-α, IFN-γ and IL-1β, which are important in the inflammatory process in RA.

Adenosine acts via four G-coupled adenosine receptors (ADOR); ADORA_1_, ADORA_2A_, ADORA_2B _and ADORA_3_. Activation of the ADORA_1 _and ADORA_2B _results in pro-inflammatory effects which include an increase in pro-inflammatory cytokine release from neutrophils and monocytes. In contrast, activation of the ADORA_2A _and ADORA_3 _results in anti-inflammatory effects with a reduction in IL-1β, TNF-α, IL-6, and decreased neutrophil superoxide production. In part, these opposing effects reflect patterns of ADOR subtype expression associated with distinct cell lineages and differentiation states, as well as linkage to divergent G protein mediated signaling pathways [2].

ADORA_3 _is highly expressed in rat synovium, peripheral blood mononuclear cells (PBMC) and lymphocytes [3]. In murine studies, MTX has been shown to exert its anti-inflammatory effects via ADORA_2A _and ADORA_3 _[4]. In MTX-treated RA patients the ADORA_3 _has been reported to be expressed at higher levels on PBMC compared to healthy controls [5]. Furthermore, expression of ADORA_2A_, ADORA_2B _and ADORA_3 _appear to be regulated by inflammatory cytokines such as TNF-α [6-8].

In an experimental model using cultured human monocytes MTX, an ADORA_1 _agonist and an ADORA_2 _antagonist were shown to enhance the formation of multi-nucleated giant cells from monocytes. The authors suggest that within the joint MTX may increase adenosine concentrations to the extent that ADORA_2 _are bound with resulting anti-inflammatory effects [9].

Whether the adenosine receptors are expressed in human joint synovial tissue, the primary lesion in RA is unknown. Furthermore, MTX through its anti-inflammatory effects may alter the expression of adenosine receptors within the synovium. We hypothesized that synovial tissue will express adenosine receptors and there will be a relationship between MTX exposure and adenosine receptor expression within the synovium. Furthermore, we hypothesized that polymorphisms within ADORA_3 _may be associated with response and/or adverse effects associated with MTX.

## Methods

Ethical approval was obtained from the Multi-Regional Ethics Committee and the Upper South B Regional Ethics Committee, New Zealand. All patients gave written informed consent obtained according to the Declaration of Helsinki. Two cohorts of patients with RA as defined by the 1987 American Rheumatism Association criteria [10] were recruited. The first cohort (*n *= 20) were used for synovial tissue gene expression studies while the second cohort (*n *= 225) were used for the ADORA_3 _genotyping. There was no overlap of the two patient cohorts.

### Synovial tissue samples - PCR and QT-PCR

Synovial tissue was obtained from the first cohort of twenty RA patients undergoing joint surgery. Total RNA was extracted from the synovial tissue using Qiagen RNeasy mini kits. Either 0.5 or 1 μg of RNA was reverse transcribed at 42°C for 50 minutes using Superscript III (Life Technologies, Carlsbad, CA USA) and Oligo (dT)_12 - 18 _primers. Expression of ADORA_1_, A_2A_, A_2B_, A_3 _and A_3variant _were examined. Quantitative real-time PCR was undertaken using TaqMan gene expression assays (Applied Biosystems, Foster City, California, USA) for ADORA_1 _(Assay ID: Hs00379752_m1), ADORA_2A _(Hs00169123_m1) and ADORA_2B _(Hs00386497_m1). We also measured three alternative transcripts produced from the ADORA_3 _gene - the ADORA_3 _assay (Hs00252933_m1) detects transcripts 1 and 3 while the ADORA_3variant _assay (Hs00181232_m1) detects transcript variant 2 only. All values were normalized to GAPDH (Hs999999905_m1).

### DNA sequencing and genotyping of variants in ADORA_3_

A second cohort consisting of 234 RA patients receiving MTX, were recruited as part of an unrelated cross-sectional study [11]. DNA was extracted from 5 ml samples of peripheral blood using phenol-chloroform [12-14]. The promoter, 5' and 3' UTRs, and the open reading frame (ORF) of the *ADORA_3 _*gene were amplified in five overlapping 680 bp to 750 bp fragments and subsequently sequenced in 15 patients with low disease activity (Disease Activity Score in 28 Joints (DAS28) < 3.2) and 15 patients with high disease activity (DAS28 ≥ 3.2). Briefly, each PCR was performed in a total volume of 20 μl containing 200 μM dNTPs, 2 mM MgCl_2_, 0.5 μM of the relevant forward and reverse primers [see Additional file 1, Table S1], 1 U of Platinum^® ^Taq DNA polymerase (Invitrogen, Carlsbad, CA, USA) and approximately 50 ng of DNA. Thermal cycling conditions comprised an initial denaturation of 94°C for two minutes, followed by 35 cycles of 94°C for 30 seconds, 60°C for 30 seconds, 72°C for one minute, and a final extension of 72°C for three minutes. Successful amplification was confirmed by analyzing 5 μl of each PCR by 1% agarose electrophoresis. Complementary DNA was purified from the remaining 15 μl of each PCR using PureLink™ PCR Purification Kit (Invitrogen) according to the manufacturer's instructions. Approximately 6 to 7 ng of purified PCR product was sequenced in both directions using BigDye^® ^Terminator Version 3.1 chemistry on an ABI 3730xl DNA Analyzer.

Genotyping of the 5'UTR SNP *rs1544224 *within ADORA_3 _was conducted using a two-tube allele-specific PCR assay. Each reaction was performed in a total volume of 10 μl containing 200 μM dNTPs, 2 mM MgCl_2_, 0.5 μM of the control primers β2Mf and β2Mr, 0.5 μM of the common forward primer ADORA_3U5f _[see Additional file 1, Table S1], 1 U of Platinum *Taq *DNA polymerase (5 U/μl) (Invitrogen) and approximately 50 ng of DNA. Reaction 1 contained 0.5 μM of primer rs1544224WT and reaction 2 contained 0.5 μM of primer rs1544224MU [see Additional file 1, Table S1]. Thermal cycling conditions comprised an initial denaturation of two minutes at 94°C, followed by 30 cycles of 30 seconds at 94°C, 30 seconds at 65°C, 30 seconds at 72°C, and a final extension of two minutes at 72°C. Sequence-validated positive controls for each genotype (*rs1544224 *C/C, C/T, T/T) were included in all genotyping runs. Reactions were visualized using 3% agarose gel electrophoresis. The primers β2Mf and β2Mr amplified a 567 bp region of the beta-2-microglobulin gene (β2M) which served as an internal control for amplification in samples where homozygosity for the SNP of interest resulted in allele-specific PCR product in only one reaction. The accuracy of the genotype was checked by repeat analysis of 5% of the samples and by sequencing PCR amplicons from a further 5% of the samples.

### Immunohistology for ADOR protein expression

Cell-specific expression of ADOR protein was investigated using two-color immunofluorescence. Primary cell-specific monoclonal antibodies (mAbs) were used in combination with goat polyclonal antibody specific for ADORA_2B _(1:50; Abcam, Cambridge, UK) as previously described [15]. For the detection of ADORA_2A _or ADORA_3_, dual labeling with mAbs was utilized, based on the following outline. However, certain combinations of cell- and ADORA-specific mAbs required that the AlexaFluor-conjugated, detection antibodies were reversed. Briefly, tissue sections were first incubated overnight at 4°C with mAbs specific for human ADORA_2A _(1:100; Lifespan Biosciences, Seattle, Washington, USA) or ADORA_3 _(1:100; Abnova, Taipei, Taiwan), diluted in RPMI-1640 (GibcoBRL, Grand Island, New York, USA)/10% FCS. Sections were washed and bound mAb was detected by incubation with AlexaFluor568-conjugated goat anti-mouse IgG (Invitrogen) for 1.5 hours diluted in RPMI/10% FCS. Sections were then sequentially washed, incubated with 1:1 PBS/10% FCS: normal mouse serum (DAKO, Glostrup, Denmark) for 30 minutes, washed, then incubated with an excess of goat anti-mouse IgG(H+L) Fab fragments (Jackson Immuno Research, Baltimore, Philadelphia, USA) for one hour before a final wash. Primary cell-specific mAbs were applied for 1.5 hours, and the sections washed again before bound primary antibody was detected with AlexaFluor488-conjugated goat anti-mouse IgG (Invitrogen) for 1.5 hours diluted in RPMI/10% FCS containing 1.25 mg/ml Hoechst 33342 nuclear stain (Molecular Probes^® ^Invitrogen, Eugene, Oregon, USA).

### Statistical analysis

The test for Hardy-Weinberg equilibrium (HWE) was performed using the internet-based program SHESIS [16]. Assessment of linkage disequilibrium between the two 5'UTR SNPs *rs1544223 *and *rs1544224 *was performed using Haploview 4.2 and HapMap Data Rel 28 Phase II + III (assembly dbSNP b126).

Data were analyzed using Graph-pad Prism 4. All data are presented as mean ± standard deviation (SD) unless otherwise stated. The statistical significance of the differences between the groups was determined by the Mann-Whitney U test and correlation was tested using the Spearman's test.

## Results

Demographics for the two patient cohorts are shown in Table 1.

**Table 1:** Demographic details of the two different patient cohorts used for genotyping and gene expression studies.

	225 patients for ADORA polymorphisms	20 patients for synovial gene expression
Age (years)	59.5 (18 to 84)	66.4 (48 to 80)
% Female	72%	90%
Disease duration (years)	10.4 (0.17 to 57)	21.1 (3.7 to 47)
Nodules	56 (24.9%)	12 (60%)
Erosions	139 (61.8%)	19 (95%)
RF +positive	178 (79.1%)	17 (85%)
CCP positive	165/214 (77.1%)	11/15 (73.3%)
Receiving MTX	225 (100%)	11 (55%)
MTX dose (mean) (mg/wk)	15.8 (5 to 25)	16.4 (7.5 to 20)
ESR	16.6 (2 to 96)	41.4 (14 to 74) (*n *= 10)
CRP	10.9 (0 to 150)	18.9 (3 to 79) (*n *= 17)
Anti-TNF therapy	0	3 (15%)
Prednisone	71 (31.6%)	6 (30%)

ADOR, adenosine receptor; CCP, cyclic citrullinated peptide; CRP, C-reactive protein; ESR, erythrocyte sedimentation rate; MTX, methotrexate; TNF, tumor necrosis factor; RF, rheumatoid factor.

### Expression of ADORA in synovial tissue

**Figure 1:**
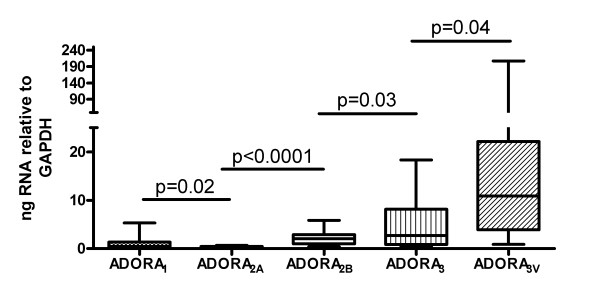
**Quantitative gene expression of ADOR in rheumatoid synovium**. ADOR, adenosine receptor.

All five ADOR genes were expressed in all 20 synovial tissues. ADORA_3variant _(A_3V_) and ADORA_3 _were the dominant ADOR subtypes present. This was followed by ADORA_2B _and ADORA_1_. ADORA_2A _was the least expressed (Figure 1). There was a significant correlation between ADORA_2A _and ADORA_2B _expression and between ADORA_3 _and ADORA_3V _expression (Table 2).

**Table 2:** Correlation between expression of different ADORA genes in rheumatoid synovium.

	ADORA_2A_	ADORA_2B_	ADORA_3_	ADORA_3V_	Combined ADORA_3_
ADORA_1_					
Spearman r	0.45	0.09	0.21	0.25	0.26
*P*	**0.04**	0.71	0.37	0.29	0.27
ADORA_2A_					
Spearman r		0.66	0.21	0.29	0.30
*P*		**0.002**	0.37	0.20	0.19
ADORA_2B_					
Spearman r			0.31	0.24	0.26
*P*			0.19	0.31	0.27
ADORA_3_					
Spearman r				0.88	
*P*				**< 0.0001**	

**Figure 2:**
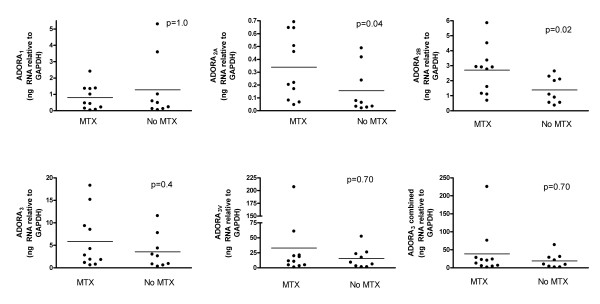
**Influence of MTX on the expression of ADOR genes in RA synovial tissue**. ADOR, adenosine receptor; MTX, methotrexate; RA, rheumatoid arthritis.

Expression of ADORA_2A _and ADORA_2B _was significantly higher in those patients receiving MTX (*n *= 11) compared to those not receiving MTX (*n *= 9). However, MTX had no apparent influence on expression of the other ADOR genes (Figure 2). There was no relationship between MTX dose and the amount of ADOR gene expression. Only three patients were receiving anti-TNF therapy without concomitant MTX. There was no significant difference in the expression of any ADOR genes between these three patients and the remaining patients (*P *> 0.05; data not shown). Similarly there was no significant difference in ADOR gene expression between those patients receiving prednisone (*n *= 6) and those not (*n *= 14) (*P *> 0.05; data not shown). There was no significant correlation between expression of any of the ADOR genes and ESR or serum CRP levels (data not shown).

### Cells expressing ADOR genes in RA synovial tissue

**Figure 3:**
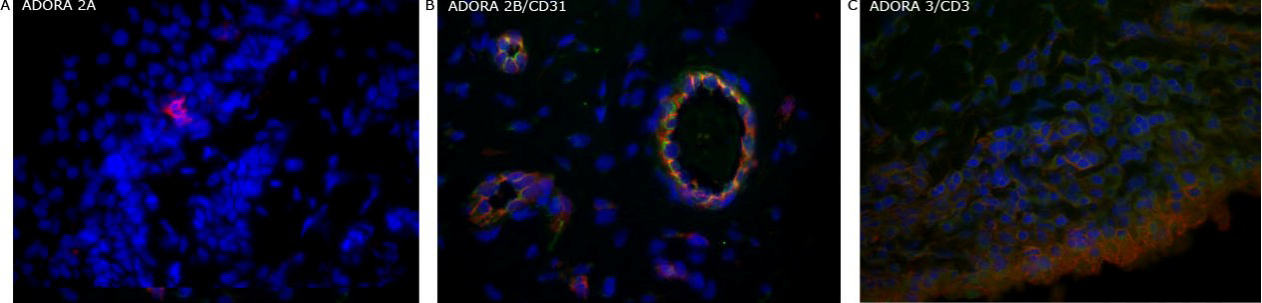
**ADORA protein expression**. Two-color immunofluorescence detecting ADORA_2A _(**A**; red), ADORA_2B _(**B**; green) and ADORA_3 _(**C**; red) protein expression in combination with cell specific markers (indicated) CD 31(B; red) and CD3 (C; green) in rheumatoid synovia. Co-localized staining appears yellow. ADOR, adenosine receptor.

Consistent with the gene expression there was widespread expression of ADOR_A3 _protein within rheumatoid synovium, co-localized to fibroblasts (Figure 3). Some, but not all infiltrating T cells stained weakly for ADOR_A3 _protein, as did interdigitating cells in the T cell areas of synovial lymphoid follicles. In contrast ADORA_2B _protein was most obvious in vascular endothelial cells (ECs) that also expressed Factor VIII (Figure 3). The ADORA_2B_-positive ECs had a cuboidal morphology, consistent with activated endothelium. However ADORA_2B _protein was also expressed by vascular ECs with a flattened morphology in osteoarthritis synovium (data not shown). ADOR_2B _protein was also expressed by fibroblasts and by some aggregated T cells in rheumatoid synovia but in amounts considerably less than was observed in ECs. Expression of ADORA_2A _protein was rare and was generally restricted to large isolated cells with a dendritic appearance. These latter cells have not been further identified. However ADOR_2A _protein was also observed in a solitary germinal center-positive lymphoid follicle, consistent with follicular dendritic cell expression.

### Sequence variants in ADORA_3_

A total of 15 SNPs were detected by sequencing *ADORA_3 _*across 15 patients with low disease activity (DAS28 ≤ 3.2) and 15 patients with high disease activity (DAS28 > 3.2) [See Additional file 2, Figure S1]. Of these polymorphisms, two were located in the promoter, four in the 5'UTR, four in the ORF, and five in the 3'UTR. All SNPs identified have been previously reported in one or more populations. Of the SNPs within the ORF, two were synonymous (*rs2789537 *and *rs2229155*) and two were non-synonymous (*rs35511654 *and *rs2800889*). Although none of the variants were found exclusively in groups with high or low disease activity, the minor allele frequencies (MAF) of the 5'UTR SNPs *rs1544223 *and *rs1544224 *were greater in the high disease activity group (MAF_high disease activity _= 0.37 versus MAF_low disease activity _= 0.13, *P *= 0.037). As a consequence, SNP *rs1544224 *was genotyped in the complete RA cohort using allele-specific PCR. The second SNP *rs1544223 *was not genotyped as it was found to be in complete linkage disequilibrium (LD) with *rs1544224 *in the subset of patients sequenced and almost complete LD (r^2 ^= 0.974) in analysis of Utah residents with Northern and Western European ancestry (CEU) and Tuscan in Italy (TSI) from the HapMap Project.

### Association between ADORA_3 _rs1544224 SNP and disease activity, and MTX toxicity

Genotyping for *rs1544224 *was successful in 96.2% of patients (225/234) and no deviation from HWE was observed (*P *= 0.43). There was no association between ADORA_3 _*rs1544224 *and any of the disease activity variables (Table 3). There was no significant association between ADORA_3 _*rs1544224 *genotype and low or high disease activity groups (*P *= 0.76). Patients homozygous for the minor allele were more likely to have 'hair loss' (*P *= 0.02) and 'forgetfulness' (*P *= 0.02), but there was no other difference in adverse effects between the genotypes [See Additional file 3, Table S2].

**Table 3:** Univariate associations between ADORA_3 _rs1544224 and disease activity.

Variable	Genotype^a^			Allelic *P *value
	11	12	22	
SJC (28)	2.9 ± 3.4	2.7 ± 3.9	3.0 ± 3.0	0.89
	123 (54.7%)	84 (37.6%)	18 (8.2%)	
TJC (28)	2.5 ± 3.5	2.4 ± 3.9	1.7 ± 2.3	0.65
	123 (54.7%)	84 (37.6%)	18 (8.2%)	
DAS28	2.9 ± 1.2	2.9 ± 1.3	2.9 ± 1.1	0.99
	123 (54.7%)	84 (37.6%)	18 (8.2%)	
CRP	1.2 ± 2.1	1.1 ± 1.4	1.1 ± 0.9	0.98
	105 (46.7%)	66 (29.3%)	16 (7.1%)	
Physicians global	23.5 ± 19.5	22.0 ± 19.8	15.5 ± 12.7	0.26
	123 (54.7%)	84 (37.6%)	18 (8.2%)	
Physicians response to MTX	22.9 ± 18.9	22.2 ± 20.7	14.5 ± 13.6	0.23
	118 (52.4%)	71 (9.3%)	18 (8.2%)	
Pain VAS	23.6 ± 22.8	22.8 ± 23.9	20.1 ± 22.9	0.83
	123 (54.7%)	84 (37.6%)	18 (8.2%)	
Patient global	26.4 ± 21.9	27.6 ± 24.6	23.7 ± 17.1	0.79
	123 (54.7%)	84 (37.6%)	18 (8.2%)	
mHAQ	0.42 ± 0.49	0.42 ± 0.45	0.37 ± 0.47	0.89
	123 (54.7%)	84 (37.6%)	18 (8.2%)	

^a^1 = major allele, 2 = minor allele of rs1544224. Top row mean ± SD, bottom row number of patients. CRP, C-reactive protein; DAS28, disease activity score in 28 joints; HAQ, Health Assessment Questionnaire; MTX, methotrexate; SD, standard deviation; SJC, swollen joint count; TJC, tender joint count; VAS, visual analog scale.

## Discussion

MTX is an effective drug in the management of RA. Its ability to suppress inflammation results, at least in part, from its ability to increase extracellular concentrations of adenosine which stimulates adenosine A_2A _and A_3 _receptors [4]. The primary site of inflammation in RA is the synovium. Suppression of the inflammatory process that drives this inflammation in the synovium and formation of the tissue destructive pannus is critical to the control of inflammatory joint symptoms and prevention of joint erosion in RA.

Most attention with respect to the anti-inflammatory mechanism of action of MTX has focused on the folate pathway with less attention on the adenosine pathway. ADORA_3 _has been reported to be expressed in PBMCs [6,17] and ADORA_1 _and A_2 _in synovial fluid cells from patients with RA [18] and isolated synoviocytes [19]. Both the adenosine A_2A _and A_3 _receptors, but not ADORA_1 _or ADORA_2B_, have been reported to be up-regulated in lymphocyte and neutrophil membranes from patients with RA as compared to healthy controls [20]. However, there have been no previous studies examining expression of the adenosine receptors within synovial tissue from patients with RA. Herein we have demonstrated that the genes for all four adenosine receptors are expressed at varying levels in rheumatoid synovium.

ADORA_3 _was expressed significantly more than the other ADOR genes. This may be the result of the inflammatory process occurring within the synovium, with previous reports suggesting that expression of ADORA_3 _is up-regulated by TNF-α via activation of NFκB [17,20]. ADORA_2A_, through which MTX is also thought to exert its anti-inflammatory effects, was expressed in the lowest amounts of all the ADOR subtypes [7].

Several possibilities could explain the difference in expression of the various adenosine receptor subtypes. These include the cellular constituents of the tissue examined. Consistent with this possibility, ADORA_3 _protein expression was more widespread in synovial tissue. In contrast ADORA_2B _protein was prominent in vascular ECs but also present in fibroblasts and some T cells, albeit in much reduced amounts; ADORA_2A _protein was restricted to cells with a dendritic appearance. Overall, the patterns of ADOR protein distribution were consistent with the hierarchical order we observed for expression of the corresponding ADOR genes. For all ADOR subtypes, we did not observe significant protein associated with the infiltrating inflammatory cells. We are not able to exclude that these patterns of protein expression and the differences in gene expression could also be affected by the inflammatory cytokine milieu within the synovium. However, we further considered the effect of anti-rheumatic therapy and the possible influence on ADORA expression.

Both MTX and anti-TNF therapy have been reported to have effects on adenosine receptor expression and affinity. MTX therapy results in up-regulation of the ADORA_2A _and ADORA_3_, but not ADORA_1 _or ADORA_2B _expression in lymphocyte and neutrophil membranes from RA patients as compared to healthy controls [20]. The up-regulation of ADORA_3 _expression on PBMCs has been observed after only ten weeks of MTX therapy [5]. In addition, the affinity of the ADORA_2A _and ADORA_3 _was lower in patients receiving MTX compared to healthy controls [20]. In comparison, in RA patients receiving anti-TNF therapy the expression and affinity of ADORA_2A _and ADORA_3 _was similar to that observed in healthy controls [20]. This is consistent with an effect of TNF or the indirect effect of inflammation in general. In our cohort, expression of ADORA_2A _and ADORA_2B _but not ADORA_3_, was increased in those patients receiving MTX compared to those not receiving MTX. While we observed no effect of anti-TNF therapy, the number of patients was small. The presence of the adenosine receptors in the synovial membrane provides evidence that MTX has the potential to exert important anti-inflammatory effects at the primary site of the inflammatory process in RA.

It is important to note that the specimens used in our study were from RA patients with late stage disease and these patients differed from the cohort used for the genotyping studies. There remains a possibility that changes in ADOR subtype expression related to MTX therapy could be different at earlier stages of disease. However, given that expression of ADORA_2A_, ADORA_2B _and ADORA_3 _is regulated by inflammatory cytokines such as TNF-α [6-8] it would be reasonable to assume there may be similar findings. This will need to be confirmed in studies of patients with early RA.

Signalling through ADOR depends on a variety of factors including receptor expression, receptor sensitivity and extra-cellular concentration of ligand. Our data show greatest gene expression of ADOR_3 _and the ADOR_3VAR _in rheumatoid synovia and that expression of a designated ADOR subtype is not necessarily exclusive to a particular cell type. Furthermore, there are known variations in the kinetic properties of the different ADOR subtypes, with ADORA_1_, ADORA_2A _and ADORA_3 _several orders of magnitude more sensitive to adenosine than ADORA_2B _[21]. It remains to be determined whether variation in expression compensates for the different kinetics attributed to the different ADOR subtypes and what consequences this has for individual synovial cell types.

The importance of ADORA_3 _in the anti-inflammatory process has also been highlighted by the finding in activated PBMCs that addition of an adenosine agonist (IB-MECA, CF101) resulted in reduced ADORA_3 _expression and down-regulation of TNF-α, thus breaking the autocrine loop and inhibiting the inflammatory process [17]. The clinical efficacy of an oral adenosine A_3 _receptor agonist, CF101, in patients with RA has been examined in a small 12-week study. The level of expression of ADORA_3 _in PBMCs at baseline correlated with ACR50 and ACR70 responses (*P *= 0.036) [22]. We have now demonstrated high levels of ADORA_3 _expression in the joint tissues targeted by the inflammation. These data provide an encouraging line of evidence for further investigation of adenosine A_3 _agonists as therapeutic agents in RA, particularly in patients with high levels of ADORA_3 _expression.

MTX has also been suggested to have a beneficial effect on cardiovascular mortality in RA, an effect that is not observed with other disease modifying anti-rheumatic drugs (DMARDs) [23,24]. This effect appears to be mediated through ADORA_2A _activation mediating reverse cholesterol transport and limiting foam cell formation [25]. One of the key challenges in the management of RA is rapid and effective disease control without provoking adverse effects. Currently there is no reliable means to predict which patients will respond to MTX, nor who will experience adverse effects. In this regard efforts have focused on the ability of polymorphisms of genes involved in the folate pathway to predict efficacy and toxicity of MTX. To date no SNP or combination of SNPs within the folate pathway has been identified that will reliably predict response or adverse effects associated with MTX.

Polymorphisms of adenosine monophosphate deaminase 1 *(AMPD1)*, aminoimidazole carboxamide ribonucleotide transformylase *(ATIC)*, and inosine triphosphate pyrophosphatase *(ITPA)*, which are involved in the adenosine pathway, have been associated with good clinical response to MTX in patients with RA in some but not all studies [26,27]. Genetic variations in the ADOR genes have received less attention but have been reported to be associated with good clinical response to MTX [26] or have no effect [27]. Polymorphisms within the ADORA_2A _gene have been associated with adverse gastrointestinal events (nausea, vomiting or diarrhea) associated with MTX in patients with RA [28]. Herein we have investigated polymorphisms within the ADORA_3 _gene. We have shown that the SNP *rs1544224 *is not associated with disease activity in patients with RA receiving MTX. Whether this SNP can predict MTX efficacy will need to be formally examined in prospective clinical studies. The cross-sectional design of this study resulted in the cohort of patients being enriched for patients who tolerated MTX. Patients with more severe adverse effects such as hepatotoxicity or myelotoxicity would have discontinued MTX and not entered this study. Nevertheless, there are a number of other adverse effects associated with MTX for which patients elect not to discontinue therapy because the benefits of therapy outweigh the adverse effects. We have shown a weak association between the ADORA_3 _*rs1544224 *SNP and forgetfulness and hair loss in our cohort. Confirmation of this will be required in other cohorts.

## Conclusions

In conclusion, we have shown that genes for adenosine receptors are expressed in RA synovium. There is differential expression of receptors such that ADORA_3 _is expressed at significantly higher levels. This evidence demonstrates the potential for MTX to exert its anti-inflammatory effects at the primary site of pathology within the joints of patients with RA.

## Abbreviations

ACR: American College of Rheumatology; ADOR: adenosine receptor; AICAR: aaminoimidazole-4-carboxamide ribonucleotide; ATIC: AICAR tyransformylase; AMPD1: adenoine monophosphate deaminase 1; cAMP: cyclic adenosine monophosphate; CRP: C-reactive protein; DAS: disease activity score; DMARD: disease modifying anti-rheumatic drug; EC: endothelial cell; ESR: erythrocyte sedimentation rate; FCS: fetal calf serum; FPGS: folylpolyglutamate synthetase; HWE: Hardy-Weinberg equilibrium; IFN: interferon; ITPA: inosine triphosphate pyrophosphatase; IL: interleukin; LD: linkage disequilibrium; mAbs: monoclonal antibodies; MAF: minor allele frequency; MTX: methotrexate; MTXGlu_n_: methotrexate polyglutamates; NFκB: nuclear factor kappa-B; ORF: open reading frame; PBMC: peripheral blood mononuclear cells; PBS: phosphate-buffered saline; PCR: polymerase chain reaction; QT-PRC: quantitative real time PCR; RA: rheumatoid arthritis; RBC: red blood cell; SD: standard deviation; SNP: single nucleotide polymorphism; TNF: tumor necrosis factor; UTR: untranslated regions.

## Competing interests

The authors declare that they have no competing interests.

## Authors' contributions

LKS contributed to the conception and design of the study, analysis and interpretation of data and writing of the manuscript. JH participated in the conception and design of the study, acquisition of data, analysis and interpretation of data, and writing of the manuscript. RLR contributed to the conception and design of the study, acquisition of data, analysis and interpretation of data, and writing of the manuscript. CF contributed to the statistical analysis and writing of the manuscript. JH participated in the analysis and interpretation of data and in revising the manuscript critically for important intellectual content. PH participated in the conception and design of study, analysis and interpretation of data, and writing of the manuscript. All authors have read and approved the final manuscript.

## Supplementary Material

Additional file 1 **Supplementary Table S1**. Primers used for amplification, sequencing, and genotyping of *ADORA_3_*. Click here for file

Additional file 2 **Supplementary Figure **1. Genomic organization of *ADORA_3_*. The *ADORA_3 _*gene is localized to chromosome 1p21-p13 and comprises two exons separated by a single large intron (not shown to scale). Untranslated regions (UTRs) and the open reading frame (ORF) are represented by light and dark grey rectangles, respectively. Location of single nucleotide polymorphisms (SNPs) found by sequencing 15 RA patients with low disease activity (DAS28 ≤ 3.2) and 15 RA patients with high disease activity (DAS28 > 3.2) are indicated by vertical arrows. Click here for file

Additional file 3 **Supplementary Table S2**. Univariate associations between ADORA_3 _rs1544224 and MTX adverse effects. Top row number of patients and bottom row percentage. Click here for file
