# Supplementary material for: Adenosine receptor expression in rheumatoid synovium: a basis for methotrexate action
Source: Arthritis Res Ther. 2012 Jun 8;14(3):R138. doi: 10.1186/ar3871 (PMC3446521; doi:10.1186/ar3871)
Supplement: Additional file 1 — Supplementary Table S1. Primers used for amplification, sequencing, and genotyping of ADORA3. [file ar3871-S1.DOC]

**Supplementary Table S1. Primers used for amplification, sequencing, and genotyping of *ADORA_3_***

| **Primer^a^** | **Sequence (5’ to 3’)** | **Product size (bp)** |
| --- | --- | --- |
| ADORA_3_ pf | AAAGTGTTTGTTAGTAAACACCAG | 750 |
| ADORA_3_ pr | ATGATAAGGAGGCAAACGGGAG |  |
| ADORA_3_U5f | AAGCAGGTGTGATGCTTCTCAGA | 680 |
| ADORA_3_U5r | TGGAAACCCTTCTCCTTAGAAAG |  |
| ADORA_3_ex1f | CTGTTTGGGGAACTAAGAGCAG | 750 |
| ADORA_3_ex1r | TTACTCAAAAAGTCTGGGCTCT |  |
| ADORA_3_ex2f | GAATTAAGAGAGTATATGCCTT | 700 |
| ADORA_3_ex2r | CTTGTGTCCAAAGAATCAGAGG |  |
| ADORA_3_U3f | TGTGCTGTACATGGGCATCCTG | 750 |
| ADORA_3_U3r | ACAGCCAGCTGGGTCTTTAAGC |  |
| rs1544224_wt | CAGAGGACAGTGCAGGATCAGGTGGGAGg**A** | 313^b^ |
| rs1544224_mu | CAGAGGACAGTGCAGGATCAGGTGGGAGg**G** |  |
| β2Mf | TGTAAACACTTGGTGCCTGATATAGCTTGA | 567^c^ |
| β2Mr | CATCAGTATCTCAGCAGGTGCCACTAATCT |  |

^a^The primers used to amplify a region were also used to sequence that region. ^b^Allele-specific reverse primers for detection of *rs1544224* were paired with ADORA3U5f. Penultimate mismatch is shown in lower case and the 3’ allele-specific base is shown in bold and underlined. ^c^Primers for 567bp of beta-microglobulin (β2M) sequence were included in both allele-specific PCRs as an internal control for amplification. Genotypes for *rs1544224* were only called for reactions in which the β2M sequence amplified.

**Supplementary Table S2: Univariate associations between ADORA_3_ rs1544224 and MTX adverse effects. Top row number of patients and bottom row percentage.**

| **Variable** | **Genotype*** | | | **Allelic p value** |
| --- | --- | --- | --- | --- |
|  | 11 | 12 | 22 |  |
| **Nausea** | 15/123 (12.2%) | 19/84  (22.6%) | 4/18  (22.2%) | 0.12 |
| **Vomiting** | 0/121 | 1/81  (1.2%) | 0/18 | 0.37 |
| **Mouth ulcers** | 31/123 (25.2%) | 24/84 (28.6%) | 6/18  (33.3%) | 0.72 |
| **Diarrhoea** | 13/123 (10.6%) | 11/84 (13.1%) | 3/18  (18.7%) | 0.70 |
| **Decreased appetite** | 17/123 (13.8%) | 14/84 (16.7%) | 5/18  (27.8%) | 0.31 |
| **Headache** | 23/123 (18.7%) | 21/84  (25%) | 6/18  (33.3%) | 0.28 |
| **Decreased concentration** | 23/123 (18.7%) | 19/84 (22.6%) | 3/18  (33.3%) | 0.74 |
| **Forgetfulness** | 32/123 (26%) | 36/84 (42.9%) | 9/18  (50%) | **0.02** |
| **Dizziness** | 17/122  (13.9%) | 10/84 (11.9%) | 4/18  (22.2%) | 0.52 |
| **Blurred vision** | 21/123 (17.1%) | 18/84 (21.4%) | 4/18  (22.2%) | 0.69 |
| **Sleep disturbance** | 16/123 (13.0%) | 20/84 (23.8%) | 4/18  (22.2%) | 0.12 |
| **Weepiness** | 21/123 (17.1%) | 17/84 (20.2%) | 4/19  (22.2%) | 0.78 |
| **Hair loss** | 1/123 (0.8%) | 6/84  (7.1%) | 2/18  (11.1%) | **0.02** |
| **Cough** | 24/123  (19.5%) | 18/84 (21.4%) | 3/18  (16.7%) | 0.88 |
| **Fever** | 6/123  (4.9%) | 4/84  (4.8%) | 1/18  (5.6%) | 0.99 |
| **Shortness of breath** | 10/123 (8.1%) | 10/84 (11.9%) | 2/18  (11.1%) | 0.66 |

*1=major allele, 2= minor allele of rs1544224
