# Supplementary figures and images for: Adenosine receptor expression in rheumatoid synovium: a basis for methotrexate action
Source: Arthritis Res Ther. 2012 Jun 8;14(3):R138. doi: 10.1186/ar3871 (PMC3446521; doi:10.1186/ar3871)

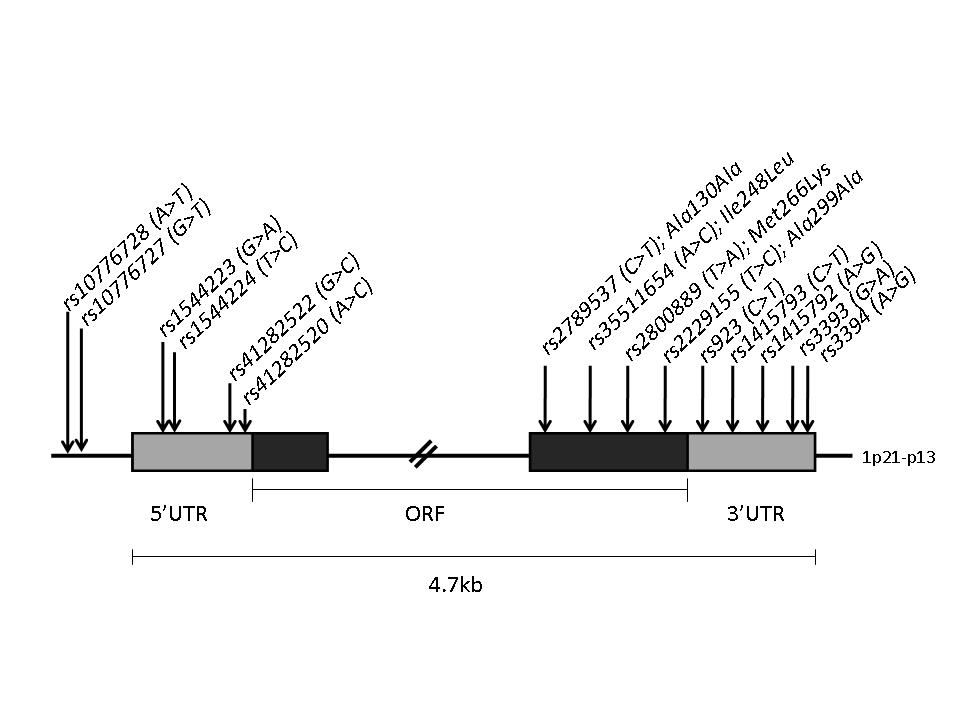

Supplement: Additional file 2 — Supplementary Figure 1. Genomic organization of ADORA3. The ADORA3 gene is localized to chromosome 1p21-p13 and comprises two exons separated by a single large intron (not shown to scale). Untranslated regions (UTRs) and the open reading frame (ORF) are represented by light and dark grey rectangles, respectively. Location of single nucleotide polymorphisms (SNPs) found by sequencing 15 RA patients with low disease activity (DAS28 ≤ 3.2) and 15 RA patients with high disease activity (DAS28 > 3.2) are indicated by vertical arrows. [file ar3871-S2.JPEG]
